# Spiders (Arachnida, Araneae) of the Visimskiy Biosphere Reserve (Middle Urals): 37 years of arachnological research

**DOI:** 10.3897/BDJ.12.e114930

**Published:** 2024-01-19

**Authors:** Nadezhda Ukhova, Sergei Esyunin, Artëm Sozontov

**Affiliations:** 1 Visimskiy State Natural Biosphere Reserve, Kirovgrad, Russia Visimskiy State Natural Biosphere Reserve Kirovgrad Russia; 2 Perm State University, Perm, Russia Perm State University Perm Russia; 3 Institute of Plant and Animal Ecology (IPAE UB RAS), Ekaterinburg, Russia Institute of Plant and Animal Ecology (IPAE UB RAS) Ekaterinburg Russia

**Keywords:** occurrence, mountain-forest belt, long-term monitoring, biodiversity, succession, pyrogenic succession, post-fire recovery, epigean spiders, litter-dwelling spiders

## Abstract

**Background:**

More than 30 articles concerning spiders’ diversity and assemblages’ structure within the Visimskiy Reserve have been published since its establishment 52 years ago. The literature provides data on 260 recorded species, one of which has been described as a new species. The majority of these records were not annotated. The peak of publication activity was in the 2^nd^ part of the 1990s and the beginning of the 21^st^ century. The greatest amount of material was collected between 2012 and 2018, within long-term plots with quantitative observations of epigean and litter-dwelling spiders, focusing on wind-throw and post-fire successions.

**New information:**

This article summarises all the literature and field primary data. We also list 18 species new to the reserve's fauna, which currently comprises 278 species. Doubtful and invalid records have been excluded from the species list. The occurrences in the dataset are supported by detailed information about vegetation cover at the time of collection. This is important in the context of research on fauna and community changes along the vegetation succession, including wind-thrown and post-fire restoration.

## Introduction

The first data on the spider fauna and communities of Visimskiy Reserve, Russia, were collected as a part of complex soil mesofauna research within the "Chronicles of Nature" programme – a special system for monitoring natural processes in ecosystems and their components (Filonov and Nukhimovskaya 1990). The Reserve's staff have been exploring the primary *Abies*-*Picea* forests and their derivative communities from 1989 to 1992. Research specifically targeting spider fauna was initiated in 1994 by the Perm State University arachnological team. Three articles devoted to the new data on the spider fauna of Urals (Esyunin et al. 1995, Esyunin and Efimik 1995, Esyunin and Efimik 1996a) included data on the spider collection available at that time and listed 148 species in total for Visimskiy Reserve. Thereafter, we prepared the first review of the spider fauna of the Reserve commemorating its 25^th^ anniversary (Ukhova and Esyunin 1996). The list of spiders was then enlarged to 164 species.

The earlier field works were carried out in an attempt to cover as a wide variety of habitats as possible to accelerate the inventory stage of the research. Wind-throw in 1995 and forest fires in 1998 and 2010 have greatly modified the structure of the Reserve vegetation. Since that time, our research has focused on the monitoring of spider associations at the model plots. We have discovered spider species new to the fauna of the Reserve during this period. Amongst them, 22 species were mentioned in ecological articles (Esyunin et al. 1996, Esyunin and Ukhova 1996, Esyunin et al. 1998, Esyunin et al. 2000, Ukhova 2001). Moreover, the new species *Theridiontigrae* Esyunin et Efimik, 1996 (Esyunin and Efimik 1996b) was described, based on the material from the Visimskiy Reserve, but later was synonymised with *Yunohamellaserpatusa* (Guan et Zhu, 1993) (Marusik and Logunov 2017).

Further, we published additions to the spider fauna every five years. The first of them contains new records of 34 species (Esyunin and Ukhova 2001), the second – nine species (Ukhova and Esyunin 2006), the third – 15 species (Esyunin and Ukhova 2011), the fourth – 21 species (Ukhova et al. 2014a, Ukhova et al. 2014b). The Reserve area was extended in 2001 (see "Geographic coverage" section) and new records came mainly from faunistic exploration of the newly-included areas. The field explorations were in 2012, 2021 and 2022 at the birch forests, which were formed after clear-cutting in the late 1960s and early 1970s. In 2013, we focused on old-growth *Betula* forests in the northern part of the Reserve (conservation areas) and young birch forests on neglected crop fields. During this period, a few species new to the fauna were listed in ecological articles (Esyunin et al. 2000, Esyunin and Efimik 2000, Esyunin et al. 2001) and faunistic reviews (Esyunin 2005, Esyunin 2007, Esyunin 2015). However, there are two spider species wrongly listed as part of the fauna because of mistypes or technical errors: the wolf spider *Pardosapalustris* (Linnaeus, 1758) (Esyunin and Efimik 2000) and the dwarf spider *Megalepthyphantesnebulosus* (Sundevall, 1830) (Esyunin and Efimik 1996a). Here, we exclude them from the list.

**Figure 1.**
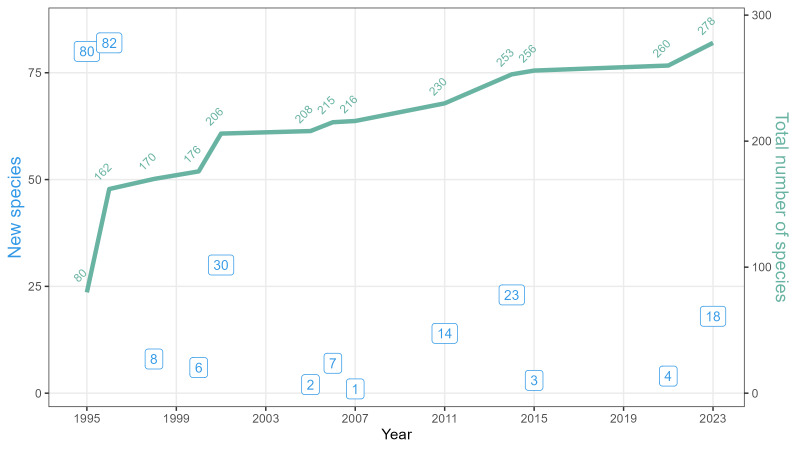
The number of spider species provided in literature: new records (blue) and total species richness of the local fauna (green).

According to the published data, there were listed 260 spider species from the Visimskiy Reserve (excepting wrong records). In this article, we provide 18 new species to the fauna which now counts 278 species in total (Table 1, Fig. 1). All the records are available openly in Darwin Core format and include information about the succession stage of sampling plots at the time of collecting.

## General description

### Purpose

Arachnological research is a part of Visimskiy Reserve research projects on the complex study of soil- and litter-living invertebrates carried out within "Chronicles of Nature" (this being a conservation monitoring programme on the condition of ecosystems and their components in protected areas). The project is devoted to inventory and long-term monitoring of the epigean invertebrate animals, including molluscs (Mollusca), carabid beetles (Carabidae), carrion beetles (Silphidae) and other taxa. The aim of the article, as a part of the project, is to summarise all literature and material data about spiders in order to obtain an up-to-date list of spider species and occurrences.

## Project description

### Title

Terrestrial invertebrates of the Visimskiy Nature Biosphere Reserve (Middle Urals)

### Personnel

Ukhova N.L., Esyunin S.L., Farzalieva G.Sh., Mazura N.S., Shumilovskikh L.S., Sozontov A.N.

### Study area description

**General description**

The Ural Mountains spread from north to south at more than 2000 km and are the border between Europe and Asia. The studied region, the Visimskiy Reserve, is located in the Middle Urals, the lowest part of the Urals. The major part of the Reserve lies on the western macroslope and only a minor part lies on the watershed crest (Prokaev and Kuznetsova 1974). The climate here is typical for the temperate continental boreal zone. The yearly average air temperature is +1.1°С and the average annual precipitation is 598.5 mm, according to data provided by the "Visim" meteorological station from 1976 to 2022.

The soddy-podzolic and brown forest soils are the most widespread soils on the Reserve territory (Gafurov and Korkina 2021). According to the forest vegetation subdivision, the Reserve belongs to the south taiga county of the Middle Ural low-mountain province of the Ural forest-mountain area (in terms of Kolesnikov (1960)). The primary vegetation is fir-spruce (*Abies* sp. and *Picea* sp.) forests, with birch (*Betula* sp.) forests being the most common derivative vegetation. Currently, the protected part of the Visimskiy Reserve encompasses 335 km^2^. A buffer zone of 461 km^2^ surrounds it. Forest cuts of different ages, mostly in the middle stages of secondary succession, predominate at this buffer. There is a service road, rarely used, along the northern reserve border, which is closed to public access. There are no permanent living buildings within the Reserve, but there are forest lodges for temporary stays. There is a small village "Bolshye Galashki" in the buffer zone, 1 km from the western Reserve border. Only three families live year-round here, with a few more residing during the summer period.

**Flora and vegetation**

The vegetation of Visimskiy Reserve is primarily forested. Forests cover 87% of the territory according to the 2000–2001 forest inventory and are represented by both boreal and nemoral types. The Reserve is situated within the mountain taiga belt, which is divided into two sub-belts. The lower one (up to 400 ± 50 m above sea level) is colder and composed of temperate boreal dark-coniferous forests. The higher sub-belt is warmer and composed of nemoral and sub-nemoral forests (Kirsanov et al. 1979). Yu.P. Gorichev (2014) explains this case of altitudinal zonation inversion in the Visimskiy Reserve by temperature inversion which is caused by sinking of cold air masses from peaks to topographic lows and accumulating there.

The most detailed forest inventory has shown 11 types of primary associations (Kolesnikov et al. 1974). However, native fir-spruce (*Abies* sp. and *Picea* sp.) forests have been altered by the industrial development of the Ural Region over the past 300 years. The remaining woodstand has been damaged in a mosaic pattern by a catastrophic wind-throw in 1995. Later, in 1998, a heavy forest fire destroyed most of the woodstand, undergrowth and herb layer in all wind-thrown areas. The fire repeated in a few wind-thrown and untouched areas. After that, there remain only three types of primary forests: 1) ferns and grasses *Abies*-*Picea* forests; 2) large-ferns *Abies*-*Picea* forests; 3) *Abies*, *Picea* and *Pinussibirica* Du Tour, 1803 forests with fern and horsetail.

Currently, primary virgin forests cover only 3% of the Reserve's territory (Sibgatullin 1987, Sibgatullin 2021) and are found on the peaks and slopes of the mountains Bolshoy Sutuk, Maliy Sutuk, Dolgiy and Kuligi. The secondary woodstand associations are mixed uneven-aged forests, dominated by *Betula* sp., *Picea* sp. and *Betula* sp. mixture or *Populustremula* L., 1753. Some of the post-fire associations with *Chamaenerionangustifolium* (L.) Scop., 1771 and *Calamagrostis* sp., located on the Maliy Sutuk Mountain, have not recovered woodstand since the fires in 1998 and 2010.

Meadows occupy no more than 1% of the territory of the Reserve. They are formed on the former forest areas harvested for crop fields and hay-making grasslands. Some of the meadows are about 300 years old. There are almost no bogs within the Reserve.

**Long-term monitoring plots**

**Figure 2.**
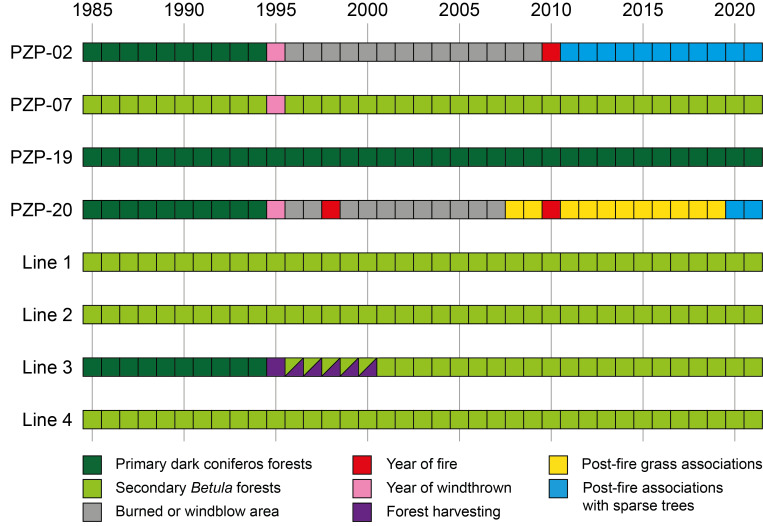
Chronology of the vegetation changes across the sampling plots.

The long-term monitoring of soil and litter invertebrate complexes covers eight permanent plots. They are established in primary ferns and grasses fir-spruce (*Abies* sp. and *Picea* sp.) forest (PZP-02 until 1995, PZP-19); in their derivative vegetation communities – post-fire associations on the various succession stages (PZP-07, PZP-20); in secondary birch (*Betula* sp.) forests formed after wood cutting (Lines 1, 2, 3 and 4). We describe all of them in detail below. Briefly, the chronology of changes in the vegetation of the plots is shown in Fig. 2.

**PZP-02** (compartment 112, sub-compartment 3) was established in 1985 in the ferns and grasses *Abies*-*Picea* forest. The plot is located 560 m above sea level (57.3960°N, 59.7419°E), on the upper part of the slightly sloping mountain "Maliy Sutuk". There are brown mountain forest soils here, middle or heavy loamy. The catastrophic wind-throw destroyed the primary forest here in 1995. Losses were about 80% of the tree stand. The sparse remaining trees dried, which led to the fire in 2010 and the permanent sampling plot was burned in a mosaic pattern, with the remaining sporadic *Betula* sp. and *Abies* sp. undergrowth only. There has been a post-fire association here since 2010, mainly formed by *Rubusidaeus* L., 1753, *Chamaenerionangustifolium* and *Calamagrostis* sp. We have registered the beginning of undergrowth recovery with sparse *Betula* sp. and *Salix* sp. trees since 2013.

**PZP-07** (compartment 123, sub-compartment 2) was established in 1991 in *Calamagrostis* and grass *Betula* forest, which itself is a derivative vegetation of primary ferns and grasses *Abies*-*Picea* forest. The plot is located 560 m above sea level (57.3884°N, 59.7430°E), on the upper part of the "Maliy Sutuk" mountain slope in the south-western direction. The microrelief is flattened here. There are brown mountain forest soils here, middle or heavy loamy. The forest damage caused by the wind-throw in 1995 was not dramatic, only 2% of trees died. The current tree stand consists of *Betula* sp., *Populustremula*, *Picea* sp. and *Abies* sp. in the ratio 5:2:2:1. The sparse undergrowth is mainly formed by *Sorbusaucuparia* L., 1753 and *Daphnemezereum* L., 1753.

**PZP-19** (compartment 112, sub-compartment 3) was established in 1996 at 570 m above sea level (57.3982°N, 59.7367°E), on the upper part of the "Maliy Sutuk" mountain slope in the north-western direction. There are brown mountain forest soils here, middle or heavy loamy. Vegetation is a primary ferns and grasses *Abies*-*Picea* forest. It includes *Picea* sp. and *Abies* sp. at a ratio of 2:1, with the addition of *Betula* sp. and *Pinussibirica*. The tree stand is sparse. The main undergrowth is *Rubusidaeus* with a mix of *Rubusmatsumuranus* H. Lév. & Vaniot. (1905), *Sambucusracemosa* L. (1753), *Ribesspicatum* E.Robson, 1796 and *Prunuspadus* L., 1753. Burned areas, wind-throws and secondary uneven-aged Betula forests surround this spot of native forest, but the primary forest on the monitoring plot was not damaged. Being almost identical to PZP-02 by soil, vegetation and hydrological conditions, PZP-19 can serve as a model plot for succession processes in such types of forests and their derivative vegetation along with PZP-02, PZP-20 and PZP-07.

**PZP-20** (compartment 112, sub-compartment 1) was established in 1998 as a model plot for wind-throw-driven and post-fire succession monitoring. The plot is located 530 m above sea level (57.4000°N, 59.7301°E) on the middle part of the "Maliy Sutuk" mountain's slight slope in the north-western direction. There are brown mountain forest soils here, middle or heavy loamy. The catastrophic wind-throw damaged a primary forest here in 1995, losses were about 80% of the tree stand. There was an intense ground fire here in 1998, which left no alive trees and only a few dry dead trunks remain. The remaining dead trees have been drying and falling gradually after that. The first stage of ecosystem restoration was the post-fire association, which included herbs and shrub vegetation. The association was formed by *Rubusidaeus*, *Chamaenerionangustifolium* and *Calamagrostis* sp. mainly with a mix of *Sambucusracemosa*, *Loniceracaerulea* L. (1753), *L.altaica* Pall. (1784) and *Rosaacicularis* Lindl., 1820. A weak ground fire happened later in 2010, which destroyed already dead trees only. Therefore, the post-fire association remained, but *Calamagrostisobtusata* Trin. (1824) and *Calamagrostislangsdorffii* Nyman (1882) increased their part within vegetation. We observed the first recovery in 2013 which was presented by single *Salixcaprea* L., 1753, *Populustremula* and *Abiessibirica* Ledeb., 1833 plantlets, but it was only on a part of the sampling plot. Over the subsequent years, elks have consistently grazed on the young undergrowth in this area, preventing the natural establishment of the tree stand. By 2018, young trees surpassed the herb layer in height and begun to form a dense canopy in certain areas. However, in 2018–2022, spider collection activities were not performed in the plot.

**Line 1** (compartment 136, sub-compartment 3) was established in 2002, on the slight slope in the intermontane area (443 m above sea level, 57.3833°N, 59.7112°E). The line is located on the forest cutting of the middle 1970s and near the river-head of the Sakalya River. Forest vegetation here is formed by *Betulapubescens* Ehrh., 1789, *Piceaobovata* Ledeb., 1833 and *Abies* sp. in a 7:2:1 ratio. The undergrowth consists of *Picea* sp., *Abies* sp. and a few *Pinussibirica*. The shrub stratum is sparse and includes *Rubusidaeus*, *Rosaacicularis* and *Prunuspadus*. The herb layer is dominated by *Calamagrostisobtusata*, with slightly less abundant *Oxalisacetosella* L. (1753), *Maianthemumbifolium* (L.) F.W.Schmidt (1794) and *Linnaeaborealis* L. (1753).

**Line 2** (compartment 150, sub-compartment 4) was established on the upper part of the Shishim Mountain, on the north-western direction of the slope (510 m above sea level, 57.3729°N, 59.6873°E). There was forest cutting here in 1975, now this is a birch forest with ferns and small grasses. The tree stand is formed by *Betula* sp., *Picea* sp. and *Abies* sp. in a 3:1:1 ratio. The undergrowth consists mostly of *Picea* sp. and only a little of *Abies* sp. The herb layer is dominated by *Dryopterisexpansa* (C.Presl) Fraser-Jenkins et Jermy (1977), *Calamagrostisobtusata* and *Oxalisacetosella*, but also *Maianthemumbifolium*, *Linnaeaborealis*, *Fragariavesca* L., 1753 and *Trientaliseuropaea* L. (1753) are present.

**Line 3** (compartment 162, sub-compartment 5) was established on the upper part of the Shishim Mountain, on the north-western direction of the slope (514 m above sea level, 57.3676°N, 59.6846°E). In the middle 1990s, there was a cutting of *Abies*-*Picea* forest. Now *Abies*-*Picea* forest with ferns and *Alliumursinum* L., 1753 is replaced by a mosaic birch forest. The tree stand is formed by *Betulapubescens*, *Pinussibirica* and *Abiessibirica* in a 7:2:1 ratio, with the addition of *Salixcaprea*, *Populustremula* and *Prunuspadus*. The sparse undergrowth is dominated by *Betula* sp., *Salix* sp. and *Sorbusaucuparia*. The shrub stratum includes *Rubusidaeus*, *Rosaacicularis* and *Lonicera* sp. The herb layer is sparse, dominated by *Deschampsiacespitosa* (L.) P. Beauv., 1812, *Calamagrostisobtusata* and *Oxalisacetosella*, with notable *Alliumvictorialis* L., 1753 in the spring season. Other herbs are less abundant in the community: *Maianthemumbifolium*, *Linnaeaborealis*, *Stellaria* sp. and *Gymnocarpiumdryopteris* (L.) Newman, 1851.

**Line 4** (compartment 163, sub-compartment 1) was established in 2012, on the upper part of the Shishim Mountain, on the north-western direction of the slope (515 m above sea level, 57.3674°N, 59.6873°E), in a mature birch forest with *Calamagrostis* sp. and little grasses. The tree stand is formed by *Betula* sp. and *Picea* sp. (in a 3:2 ratio), with the same species in the second forest stratum. The sparse undergrowth consists of *Sorbusaucuparia* and *Rosaacicularis*. The herb and subshrub layer is dominated by *Oxalisacetosella*, *Gymnocarpiumdryopteris*, *Fragariavesca*, *Calamagrostisobtusata* and *Stellaria* sp. Other species are less abundant in the community: *Vacciniummyrtillus* L., 1753, *Alliumvictorialis*, *Trientaliseuropaea*, *Linnaeaborealis* and various fern species.

### Design description

The data are based mainly on the complex soil- and litter-living invertebrates research, which was performed at eight permanent sampling plots (see the "Study area description" section and the "Long-term monitoring plots" subsection). This source has produced about 3/4 of the occurrences. Each sample has a brief landscape-geographical description and vegetation relevé. Due to the obvious succession on the permanent sampling plots, the provided descriptions reflect the relevant succession stage at the time of collecting spiders. The last 1/4 occurrences were obtained as an addition from temporary sampling plots and sporadic spider collecting. The vast majority of records are georeferenced and have metadata such as date, altitude, habitats (including a succession stage, if available), collecting method and sampling effort, so they can be used in quantitative ecological research. Weather data (absent in the dataset, but available upon request) comes from field journals and the "Visim" meteorological station.

## Sampling methods

### Study extent

Pitfall traps, litter sifting, entomological net-sweeping, tree and shrub crown shaking, manual collecting.

### Sampling description

All data about epigeobiont, soil- and litter-dwelling spiders come from pitfall traps and litter sifting. Pitfall traps were usually installed in a line of 10 (from 4 to 11). Their duration was typically 1–2 weeks (from 3 days to 3 weeks). Glass cans (an opening diameter of 75 mm) or plastic glasses (an opening diameter of 65 mm) filled with formaldehyde or acetic acid solution were used as traps. We sampled soil litter from the surfaces of 50 × 50, 20 × 20 or 15 × 15 cm, sifted it and investigated it on white plastic film. Spiders of the herb layer were collected by entomological net-sweeping. Quantitative samples come from five replications by 20 sweeps, unless otherwise defined in the `samplingEffort` field. A small portion of faunistic material was picked by tree or shrub crown-shaking and by manual collecting.

### Quality control

The collection is stored at the Perm State University (abbreviated as "PSU" in the dataset). Esyunin S.L. has identified all the adult individuals to the species level. Juvenile individuals were identified to the species, genera or family level depending on the informative value of morphological features (body size, shape and colouration, eye configuration, chaetotaxy etc.). The taxonomical nomenclature accords with the World Spider Catalog (2023).

### Step description

The project is long-term and continues.

## Geographic coverage

### Description

**Figure 3.**
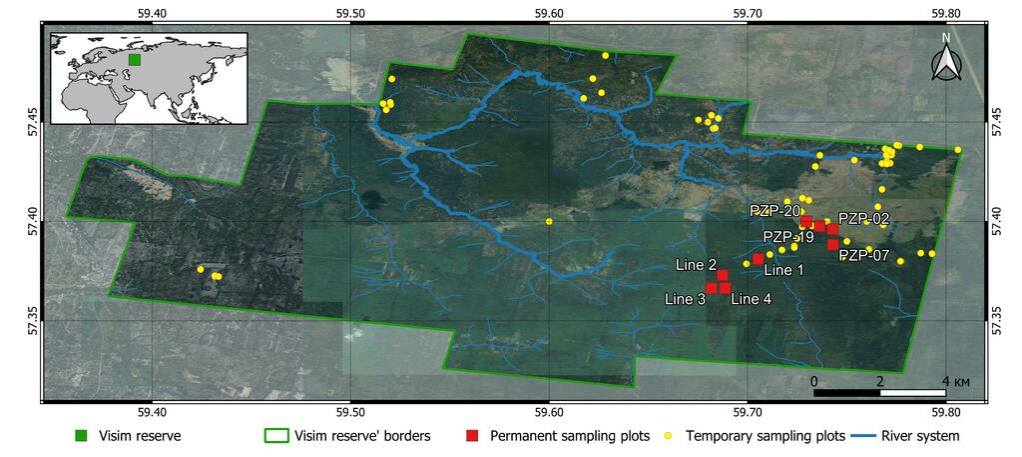
Map of the Visimskiy Reserve and sampling plots.

The Visimskiy Biosphere Reserve is located in the Sverdlovsk Region and captures three city districts: Gornouralskiy, Kirovgradskiy and Verkhniy Tagil (Fig. 3). Originally, in 1971, the Reserve area was 9,531 ha (95 km^2^), being extended in 1973, 1980 and 2001, currently accounting 33,497 ha (335 km^2^). The territory is elongated east to west. Its main part is situated on the western macroslope of the Ural Mountain Range, upstream to the Sulyom River. A smaller part is located on the eastern macroslope and includes the watershed and upstream of the Vogulka River.

### Coordinates

57.37 and 57.48 Latitude; 59.42 and 59.81 Longitude.

## Taxonomic coverage

### Description

The dataset includes occurrences of 278 spider species (146 genera and 20 families in total) (Table 1). Of them, we list 18 species for the first time: *Agnyphantesexpunctus* (O.Pickard-Cambridge, 1875), *Agroecalusatica* (L.Koch, 1875), *Argyronetaaquatica* (Clerck, 1757), *Cheiracanthiumpunctorium* (Villers, 1789), *Clubionapallidula* (Clerck, 1757), *Dictynamajor* Menge, 1869, *Entelecaracongenera* (O.Pickard-Cambridge, 1879), *Euophrysfrontalis* (Walckenaer, 1802), *Haplodrassusumbratilis* (L.Koch, 1866), *Improphantescomplicatus* (Emerton, 1882), *Lophommapunctatum* (Blackwall, 1841), *Pardosaprativaga* (L.Koch, 1870), *Talaverathorelli* (Kulczyński, 1891), *Tetragnathadearmata* Thorell, 1873, *Thyreosteniusparasiticus* (Westring, 1851), *Walckenaeriakochi* (O.Pickard-Cambridge, 1873), *Xysticuskochi* Thorell, 1872 and *Zeloteselectus* (C.L.Koch, 1839). We also exclude two previously recorded species from this list (*Pardosapalustris* (Linnaeus, 1758) (Esyunin and Efimik 2000) and *Megalepthyphantesnebulosus* (Sundevall, 1830) (Esyunin and Efimik 1996a)), as wrongly provided because of mistypes and inexact georeferencing.

### Taxa included

**Table:** 

Rank	Scientific Name	Common Name
order	Araneae	Spiders

## Traits coverage

### Data coverage of traits

**Figure 4.**
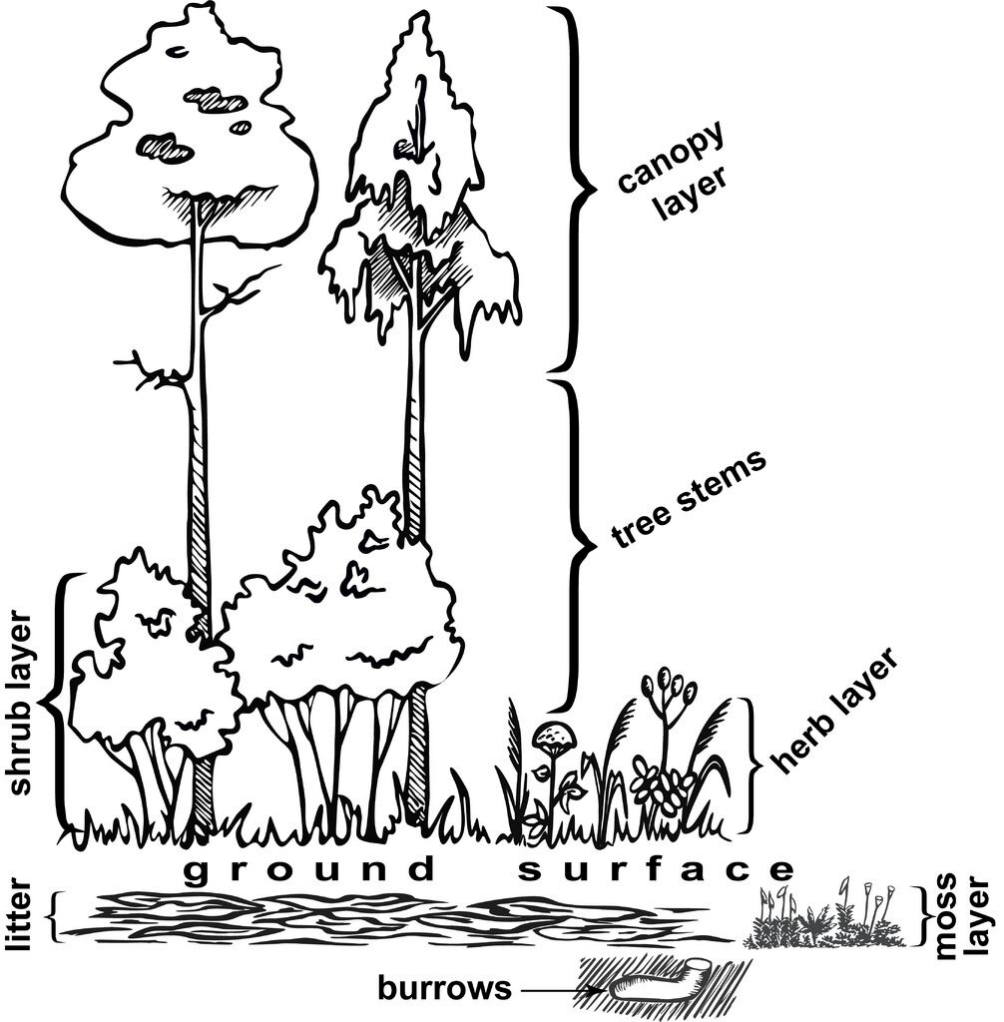
The scheme of vegetation and other layers preferable by spiders (by Sozontov and Esyunin (2022): P. 17)

The listed species are accompanied by such traits as a range type and a preferable vegetation layer (Table 1). The range type is given in two columns: a longitudinal range group (general, first term) + a longitudinal range type (detailed, second term in brackets) in the first one and a latitudinal (zonal) range type in the second, respectively. The preferable vegetation layer is given according to the monograph (Sozontov and Esyunin 2022: P. 16–17) and can be one or more of the following: litter, moss layer, ground surface, herb layer, shrub layer, tree stems and canopy layer (Fig. 4), with additional specification if available.

Selecting a range types classification, we follow a paradigm viewing a range as a combination of a few components. Traditionally, two components are spatial extension in the west-east and the north-south direction (zonal expansion). The third component, altitudinal extension, was suggested by K.B. Gorodkov (1984) on the example of insects' ranges. Such an approach allows not to mix heterogeneous distributional factors, but to analyse range components separately. Based on this paradigm, the principles of typology of the Palearctic and Holarctic spider ranges were suggested in articles (Esyunin et al. 2010, Esyunin and Marusik 2011) where the importance of hierarchy for a classification was accentuated. Later, we specified the scheme and defined the considered classes (Sozontov and Esyunin 2022: P. 16–20). Our early review did not include the species whose ranges extend far to the north or into the mountains. For such species, we provide additional descriptions of the latitudinal (zonal) components of their ranges.

- **Arcto-boreal** – species occur in both the tundra (with a forest-tundra subzone) and boreal forest zones.
- **Boreal-mountain** – species occur in both the boreal forest zone and mountain region (i.e. in mountain forest and mountain tundra belts).
- **Arcto-boreal-mountain** – species occur in the tundra (with forest-tundra subzone), the boreal forest zones and in mountain regions (i.e. in mountain forest and mountain tundra belts).

## Temporal coverage

### Notes

1984-08-16 through 2022-06-28

## Usage licence

### Usage licence

Other

### IP rights notes

This work is licensed under a Creative Commons Attribution (CC-BY 4.0) License.

## Data resources

### Data package title

Spiders (Arachnida: Araneae) of the Visimskiy Nature Biosphere Reserve (Middle Urals)

### Resource link

https://doi.org/10.15468/yt9r4q

### Alternative identifiers

https://www.gbif.org/dataset/f0bfae70-680e-4834-9dde-0d97507ef16b

### Number of data sets

1

### Data set 1.

#### Data set name

Spiders (Arachnida: Araneae) of the Visimskiy Nature Biosphere Reserve (Middle Urals)

#### Data format

Darwin Core

#### Download URL

http://gbif.ru:8080/ipt/resource?r=visim_spiders

#### Description

The dataset (Sozontov et al. 2023) consists of 6,408 records of spiders’ occurrences from the Visimskiy Nature Biosphere Reserve (Middle Urals), accounting for 278 species of 146 genera and 28,460 individuals identified to the species level. The vast majority of records are georeferenced and have such information as date, altitude, habitats (including a succession stage, if available), collecting method and sampling effort, which makes them suitable for quantitative ecological research. Each permanent sampling plot can be easily linked to the brief landscape-geographical description and vegetation relevé, reflecting the relevant succession stage at the time of spider collecting.

**Data set 1.** 

Column label	Column description
type	The nature or genre of the resource. A variable (two terms: "Event" and "PhysicalObject").
modified	The most recent date-time on which the resource was changed. A constant ("2023").
language	Thelanguage of the resource. A constant ("en" = English).
license	A legal document giving official permission to do something with the resource. A constant ("CC BY 4.0").
rightsHolder	A person or organisation owning or managing rights over the resource. A constant ("Institute of Plant and Animal Ecology (IPAE), UB RAS").
institutionCode	The name (or acronym) in use by the institution having custody of the object(s) or information referred to in the record. A constant ("Institute of Plant and Animal Ecology (IPAE), UB RAS").
datasetName	The name identifying the dataset from which the record was derived. A constant ("Spiders (Arachnida: Araneae) of the Visimskiy Nature Biosphere Reserve (Middle Urals)").
basisOfRecord	The specific nature of the data record. A variable (two terms: "HumanObservation" and "PreservedSpecimen").
occurrenceID	An identifier for the dwc:Occurrence (as opposed to a particular digital record of the dwc:Occurrence). In the absence of a persistent global unique identifier, construct one from a combination of identifiers in the record that will most closely make the dwc:occurrenceID globally unique. A variable.
catalogNumber	An identifier (preferably unique) for the record within the dataset or collection. A variable.
recordedBy	A person, group or organisation responsible for recording the original dwc:Occurrence. A variable.
occurrenceStatus	A statement about the presence or absence of a dwc:Taxon at a dcterms:Location. A constant ("present").
disposition	The current state of a dwc:MaterialEntity with respect to a collection. A variable ("in collection" or emtpy).
associatedTaxa	A list (concatenated and separated) of identifiers or names of dwc:Taxon records and the associations of this dwc:Occurrence to each of them. A variable.
eventDate	The date-time or interval during which a dwc:Event occurred. For occurrences, this is the date-time when the dwc:Event was recorded. Not suitable for a time in a geological context. A variable.
startDayOfYear	The earliest integer day of the year on which the dwc:Event occurred (1 for January 1, 365 for December 31, except in a leap year, in which case it is 366). A variable.
endDayOfYear	The latest integer day of the year on which the dwc:Event occurred (1 for January 1, 365 for December 31, except in a leap year, in which case it is 366). A variable.
year	The four-digit year in which the dwc:Event occurred, according to the Common Era Calendar. A variable.
month	The integer month in which the dwc:Event occurred. A variable.
day	The integer day of the month on which the dwc:Event occurred. A variable.
habitat	A category or description of the habitat in which the dwc:Event occurred. A variable.
eventRemarks	Comments or notes about the dwc:Event. A variable.
samplingProtocol	The names of, references to, or descriptions of the methods or protocols used during a dwc:Event. A variable.
samplingEffort	The amount of effort expended during a dwc:Event. A variable.
higherGeography	A list (concatenated and separated) of geographic names less specific than the information captured in the dwc:locality term. A constant ("Urals | Middle Urals").
continent	The name of the continent in which the dcterms:Location occurs. A variable ("Europe" or "Asia").
country	The name of the country or major administrative unit in which the dcterms:Location occurs. A constant ("Russian Federation").
countryCode	The standard code for the country in which the dcterms:Location occurs. A constant ("RU").
stateProvince	The name of the next smaller administrative region than country (state, province, canton, department, region etc.) in which the dcterms:Location occurs. A constant ("Sverdlovsk Area").
locality	The specific description of the place. A variable.
minimumElevationInMeters	Minimum Elevation In metres. A variable.
maximumElevationInMeters	Maximum Elevation In meters. A variable.
verbatimCoordinates	The verbatim original spatial coordinates of the dcterms:Location. The coordinate ellipsoid, geodeticDatum, or full Spatial Reference System (SRS) for these coordinates should be stored in dwc:verbatimSRS and the coordinate system should be stored in dwc:verbatimCoordinateSystem. A variable.
decimalLatitude	Decimal Latitude. A variable.
decimalLongitude	Decimal Longitude. A variable.
geodeticDatum	The ellipsoid, geodetic datum or spatial reference system (SRS), upon which the geographic coordinates given in dwc:decimalLatitude and dwc:decimalLongitude are based. A constant ("WGS84").
coordinateUncertaintyInMeters	The horizontal distance (in metres) from the given dwc:decimalLatitude and dwc:decimalLongitude describing the smallest circle containing the whole of the dcterms:Location. Leave the value empty if the uncertainty is unknown, cannot be estimated or is not applicable (because there are no coordinates). Zero is not a valid value for this term. A variable.
georeferencedBy	A list (concatenated and separated) of names of people, groups or organisations who determined the georeference (spatial representation) for the dcterms:Location. A constant ("Ukhova N.L.").
georeferencedDate	The date on which the dcterms:Location was georeferenced. A variable.
identifiedBy	A list (concatenated and separated) of names of people, groups or organisations who assigned the dwc:Taxon to the subject. A variable.
dateIdentified	The date on which the subject was determined as representing the dwc:Taxon. A variable.
individualCount	The number of individuals present at the time of the dwc:Occurrence. A variable.
sex	The sex of the biological individual(s) represented in the dwc:Occurrence. A variable.
lifeStage	The age class or life stage of the dwc:Organism(s) at the time the dwc:Occurrence was recorded. A variable.
scientificName	The full scientific name, with authorship and date information, if known. When forming part of a dwc:Identification, this should be the name in lowest level taxonomic rank that can be determined. This term should not contain identification qualifications, which should instead be supplied in the dwc:identificationQualifier term. A variable.
family	The full scientific name of the family in which the dwc:Taxon is classified. A variable.
genus	The full scientific name of the genus in which the dwc:Taxon is classified. A variable.
specificEpithet	The name of the first or species epithet of the dwc:scientificName. A variable.
scientificNameAuthorship	The authorship information for the dwc:scientificName formatted according to the conventions of the applicable dwc:nomenclaturalCode. A variable.
taxonRank	The taxonomic rank of the most specific name in the dwc:scientificName. A constant ("SPECIES").
kingdom	The full scientific name of the kingdom in which the dwc:Taxon is classified. A constant ("Animalia").
order	The full scientific name of the order in which the dwc:Taxon is classified. A constant ("Araneae").
collectionCode	The name, acronym, coden or initialism identifying the collection or dataset from which the record was derived. A constant ("PSU").
parentEventID	An identifier for the broader dwc:Event that groups this and potentially other dwc:Events. A variable.
eventID	An identifier for the set of information associated with a dwc:Event (something that occurs at a place and time). May be a global unique identifier or an identifier specific to the dataset. A variable.
coordinatePrecision	A decimal representation of the precision of the coordinates given in the dwc:decimalLatitude and dwc:decimalLongitude. A variable.
organismQuantity	A number or enumeration value for the quantity of dwc:Organisms. A variable.
organismQuantityType	The type of quantification system used for the quantity of dwc:Organisms (in the "organismQuantity" field). A variable ("individuals per 100 net sweeps" or "individuals per 100 traps-days").

## Figures and Tables

**Table 1.** List of spider species of the Visimskiy Reserve.

**Family**	**Species**	**Range group (range type)**	**Range zonal**	**Stratum**	**First record**
Araneidae	*Araneusalsine* (Walckenaer, 1802)	Palaearctic (Trans-Palaearctic)	Temperate	herb, shrub	Ukhova and Esyunin (1996)
Araneidae	*Araneusangulatus* Clerck, 1757	Palaearctic (West-Central-Palaearctic)	Temperate	canopy	Esyunin and Efimik (1996a)
Araneidae	*Araneusdiadematus* Clerck, 1757	Holarctic (Circum-Holarctic)	Temperate	shrub, canopy	Ukhova et al. (2014a)
Araneidae	*Araneusmarmoreus* Clerck, 1757	Holarctic (Circum-Holarctic)	Temperate	herb, shrub, canopy	Esyunin and Efimik (1996a)
Araneidae	*Araneusnordmanni* (Thorell, 1870)	Holarctic (Circum-Holarctic)	Boreal	canopy	Esyunin and Efimik (1996a)
Araneidae	*Araneusquadratus* Clerck, 1757	Palaearctic (West-Central-Palaearctic)	Polyzonal	herb	Esyunin and Efimik (1996a)
Araneidae	*Araneussaevus* (L. Koch, 1872)	Holarctic (Circum-Holarctic)	Temperate	canopy	Ukhova et al. (2014a)
Araneidae	*Araneussturmi* (Hahn, 1831)	Palaearctic (West-Central-Palaearctic)	Temperate	shrub, canopy	Esyunin and Efimik (1996a) (as *Atea*)
Araneidae	*Araniellaproxima* (Kulczyński, 1885)	Holarctic (Circum-Holarctic)	Temperate	herb, shrub, canopy	Esyunin and Efimik (1996a)
Araneidae	*Cercidiaprominens* (Westring, 1851)	Holarctic (Circum-Holarctic)	Temperate	herb	Esyunin and Ukhova (2001)
Araneidae	*Cyclosaconica* (Pallas, 1772)	Holarctic (Circum-Holarctic)	Temperate	canopy	Esyunin and Efimik (1996a)
Araneidae	*Gibbaraneaomoeda* (Thorell, 1870)	Palaearctic (Trans-European Siberian)	Boreal-mountain	canopy	Ukhova et al. (2014a)
Araneidae	*Hypsosingasanguinea* (C. L. Koch, 1844)	Palaearctic (Trans-Palaearctic)	Temperate	herb	Esyunin and Ukhova (2001)
Araneidae	*Larinioidescornutus* (Clerck, 1757)	Holarctic (Circum-Holarctic)	Temperate	herb, shrub	Ukhova and Esyunin (1996)
Araneidae	*Larinioidespatagiatus* (Clerck, 1757)	Holarctic (Circum-Holarctic)	Polyzonal	shrub, canopy	Esyunin and Efimik (1996a)
Araneidae	*Mangoraacalypha* (Walckenaer, 1802)	Palaearctic (West-Central-Palaearctic)	Subboreal	herb	Esyunin and Ukhova (2011)
Cheiracanthidae	*Cheiracanthiumerraticum* (Walckenaer, 1802)	Palaearctic (Trans-Palaearctic)	Subboreal	herb	Esyunin and Efimik (1996a)
Cheiracanthidae	*Cheiracanthiumpunctorium* (Villers, 1789)	Palaearctic (West-Central-Palaearctic)	Subboreal	herb	**New data**
Clubionidae	*Clubionacaerulescens* L. Koch, 1867	Palaearctic (Trans-Palaearctic)	Temperate	ground, herb	Esyunin and Efimik (1996a)
Clubionidae	*Clubionagermanica* Thorell, 1871	Palaearctic (West-Central-Palaearctic)	Temperate	herb	Esyunin and Ukhova (2001)
Clubionidae	*Clubionakulczynskii* Lessert, 1905	Holarctic (Circum-Holarctic)	Temperate	herb, shrub	Esyunin and Efimik (1996a)
Clubionidae	*Clubionalutescens* Westring, 1851	Palaearctic (Trans-Palaearctic)	Temperate	shrub, tree stems, canopy	Esyunin and Ukhova (2001)
Clubionidae	*Clubionapallidula* (Clerck, 1757)	Palaearctic (Trans-Palaearctic)	Temperate	canopy	**New data**
Clubionidae	*Clubionareclusa* O. Pickard-Cambridge, 1863	Palaearctic (Trans-Palaearctic)	Temperate	herb	Ukhova et al. (2014a)
Clubionidae	*Clubionasubtilis* L. Koch, 1867	Palaearctic (Trans-Palaearctic)	Temperate	litter, moss, herb	Esyunin (2015)
Cybaeidae	*Cryphoecasilvicola* (C. L. Koch, 1834)	Palaearctic (Trans-Palaearctic)	Temperate	litter, tree stems	Esyunin and Efimik (1995)
Dictynidae	*Argyronetaaquatica* (Clerck, 1757)	Palaearctic (Trans-Palaearctic)	Polyzonal	aquatic	**New data**
Dictynidae	*Dictynaarundinacea* (Linnaeus, 1758)	Holarctic (Circum-Holarctic)	Polyzonal	herb, shrub	Esyunin and Efimik (1996a)
Dictynidae	*Dictynamajor* Menge, 1869	Holarctic (Circum-Holarctic)	Temperate	herb, shrub, canopy	**New data**
Dictynidae	*Dictynapusilla* Thorell, 1856	Palaearctic (Trans-Palaearctic)	Temperate	herb, shrub, canopy	Esyunin and Efimik (1995)
Gnaphosidae	*Drassodeslapidosus* (Walckenaer, 1802)	Palaearctic (Trans-Palaearctic)	Temperate	ground, under stones	Esyunin and Efimik (1996a)
Gnaphosidae	*Drassodespubescens* (Thorell, 1856)	Palaearctic (Trans-Palaearctic)	Temperate	ground, under stones	Esyunin and Ukhova (2001)
Gnaphosidae	*Drassylluslutetianus* (L. Koch, 1866)	Palaearctic (European-West Siberian)	Temperate	litter, ground	Ukhova et al. (2014a)
Gnaphosidae	*Drassylluspraeficus* (L. Koch, 1866)	Palaearctic (West-Palaearctic)	Temperate	litter, ground	Ukhova and Esyunin (2006)
Gnaphosidae	*Drassylluspusillus* (C. L. Koch, 1833)	Palaearctic (Trans-Palaearctic)	Temperate	litter, ground	Esyunin and Ukhova (2011)
Gnaphosidae	*Gnaphosamontana* (L. Koch, 1866)	Palaearctic (European-West Siberian)	Temperate	ground, rocks	Ukhova and Esyunin (1996)
Gnaphosidae	*Gnaphosamuscorum* (L. Koch, 1866)	Holarctic (Circum-Holarctic)	Boreal-mountain	ground	Ukhova et al. (2014a)
Gnaphosidae	*Haplodrassuscognatus* (Westring, 1861)	Palaearctic (Amphi-Palaearctic)	Temperate	litter, ground	Esyunin and Ukhova (2001)
Gnaphosidae	*Haplodrassusmoderatus* (Kulczyński, 1897)	Palaearctic (Trans-European Siberian)	Temperate	litter, ground	Ukhova and Esyunin (2006)
Gnaphosidae	*Haplodrassussignifer* (C. L. Koch, 1839)	Holarctic (Circum-Holarctic)	Polyzonal	litter, ground	Esyunin and Ukhova (2001)
Gnaphosidae	*Haplodrassussilvestris* (Blackwall, 1833)	Palaearctic (European)	Subboreal	litter, ground	Ukhova et al. (2014a)
Gnaphosidae	*Haplodrassussoerenseni* (Strand, 1900)	Palaearctic (Trans-Palaearctic)	Polyzonal	litter, ground	Esyunin and Efimik (1996a)
Gnaphosidae	*Haplodrassusumbratilis* (L. Koch, 1866)	Palaearctic (European-West Siberian)	Subboreal	litter, ground	**New data**
Gnaphosidae	*Micariaaenea* Thorell, 1871	Holarctic (Circum-Holarctic)	Boreal-mountain	litter, ground	Ukhova et al. (2014a)
Gnaphosidae	*Micarianivosa* L. Koch, 1866	Palaearctic (European-West Siberian)	Temperate	litter, ground	Ukhova et al. (2014a)
Gnaphosidae	*Micariapulicaria* (Sundevall, 1831)	Holarctic (Circum-Holarctic)	Temperate	litter, ground	Esyunin and Efimik (1996a)
Gnaphosidae	*Micariasilesiaca* L. Koch, 1875	Palaearctic (European-West Siberian)	Temperate	litter, ground	Ukhova et al. (2014a)
Gnaphosidae	*Micariasubopaca* Westring, 1861	Palaearctic (Trans-Palaearctic)	Temperate	litter, ground	Esyunin and Ukhova (2011) (as *Arboricaria*)
Gnaphosidae	*Zelotesazsheganovae* Esyunin & Efimik, 1992	Palaearctic (Amphi-Palaearctic)	Subboreal	litter, ground	Ukhova and Esyunin (2006)
Gnaphosidae	*Zelotesclivicola* (L. Koch, 1870)	Palaearctic (European-West Siberian)	Temperate	litter, ground	Esyunin and Ukhova (2001)
Gnaphosidae	*Zeloteselectus* (C. L. Koch, 1839)	Palaearctic (West-Central-Palaearctic)	Subboreal-subarid	litter, ground	**New data**
Gnaphosidae	*Zeloteslatreillei* (Simon, 1878)	Palaearctic (West-Palaearctic)	Temperate	litter, ground	Esyunin and Ukhova (2011)
Gnaphosidae	*Zelotessubterraneus* (C. L. Koch, 1833)	Palaearctic (West-Central-Palaearctic)	Polyzonal	litter, ground	Esyunin and Efimik (1996a)
Hahniidae	*Antisteaelegans* (Blackwall, 1841)	Palaearctic (European-West Siberian)	Boreal	litter, moss	Esyunin and Ukhova (2001)
Hahniidae	*Hahniaononidum* Simon, 1875	Holarctic (Circum-Holarctic)	Polyzonal	litter, ground	Esyunin et al. (1998)
Hahniidae	*Hahniapusilla* C. L. Koch, 1841	Palaearctic (European-West Siberian)	Temperate	litter	Esyunin and Efimik (1995)
Hahniidae	*Mastigusaarietina* (Thorell, 1871)	Palaearctic (European-West Siberian)	Subboreal	litter	Esyunin and Ukhova (2001)
Linyphiidae	*Abiskoaabiskoensis* (Holm, 1945)	Palaearctic (North European-Trans-Siberian)	Boreal	herb, tree stems	Esyunin et al. (1995) (as *Lepthyphantes*)
Linyphiidae	*Agnyphantesexpunctus* (O. Pickard-Cambridge, 1875)	Palaearctic (Trans-European Siberian)	Boreal-mountain	herb, canopy	**New data**
Linyphiidae	*Agynetaaffinis* (Kulczyński, 1898)	Palaearctic (Trans-Palaearctic)	Temperate	litter	Esyunin et al. (1995) (as *A.beata*)
Linyphiidae	*Agynetaallosubtilis* Loksa, 1965	Holarctic (Sub-Circum-Holarctic)	Temperate	litter, moss	Esyunin et al. (1995) (as *A.subtilis*)
Linyphiidae	*Agynetaconigera* (O. Pickard-Cambridge, 1863)	Palaearctic (Trans-Palaearctic)	Temperate	litter, moss	Esyunin et al. (1995)
Linyphiidae	*Agynetadecora* (O. Pickard-Cambridge, 1871)	Holarctic (Sub-Circum-Holarctic)	Temperate	litter, moss	Ukhova et al. (2014a)
Linyphiidae	*Agynetagulosa* (L. Koch, 1869)	Palaearctic (Trans-European Siberian)	Boreal-mountain	litter, moss	Esyunin and Ukhova (2011)
Linyphiidae	*Agynetainnotabilis* (O. Pickard-Cambridge, 1863)	Palaearctic (European)	Subboreal	tree stems	Ukhova et al. (2014b)
Linyphiidae	*Agynetamollis* (O. Pickard-Cambridge, 1871)	Holarctic (Circum-Holarctic)	Polyzonal	litter	Esyunin and Ukhova (2001)
Linyphiidae	*Agynetamossica* (Schikora, 1993)	Palaearctic (European-Middle Siberian)	Arcto-boreal	litter, moss	Esyunin and Ukhova (2001) (as *A.saxatilis*)
Linyphiidae	*Agynetaolivacea* (Emerton, 1882)	Holarctic (Circum-Holarctic)	Boreal-mountain	litter	Esyunin and Efimik (1996a)
Linyphiidae	*Agynetaramosa* Jackson, 1912	Palaearctic (European-West Siberian)	Boreal	litter, moss	Esyunin and Ukhova (2001)
Linyphiidae	*Agynetarurestris* (C. L. Koch, 1836)	Palaearctic (West-Palaearctic)	Polyzonal	litter	Esyunin et al. (1995) (female only)
Linyphiidae	*Agynetatibialis* Tanasevitch, 2005	Palaearctic (North European-West Siberian)	Boreal-mountain	litter	Esyunin (2007)
Linyphiidae	*Allomengeascopigera* (Grube, 1859)	Holarctic (Circum-Holarctic)	Boreal-mountain	ground, herb	Esyunin and Efimik (1996a)
Linyphiidae	*Anguliphantesangulipalpis* (Westring, 1851)	Palaearctic (European-West Siberian)	Temperate	litter	Esyunin et al. (1995) (as Lepthyphantes)
Linyphiidae	*Araeoncushumilis* (Blackwall, 1841)	Palaearctic (West-Palaearctic)	Temperate	litter, ground	Esyunin et al. (2021)
Linyphiidae	*Asthenarguspaganus* (Simon, 1884)	Palaearctic (European-West Siberian)	Temperate	litter, moss	Esyunin et al. (1995)
Linyphiidae	*Baryphymatrifrons* (O. Pickard-Cambridge, 1863)	Holarctic (Circum-Holarctic)	Temperate	herb, shrub	Esyunin and Ukhova (2001) (as *Minyrioloides*)
Linyphiidae	*Bathyphantesgracilis* (Blackwall, 1841)	Holarctic (Circum-Holarctic)	Polyzonal	litter, moss	Ukhova and Esyunin (2006)
Linyphiidae	*Bathyphantesnigrinus* (Westring, 1851)	Palaearctic (European-West Siberian)	Temperate	litter, herb	Esyunin and Efimik (1996a)
Linyphiidae	*Bathyphantessetiger* F. O. Pickard-Cambridge, 1894	Palaearctic (Trans-European Siberian)	Boreal	litter	Ukhova et al. (2014b)
Linyphiidae	*Bolyphantesalticeps* (Sundevall, 1833)	Palaearctic (Trans-Palaearctic)	Temperate	herb	Esyunin et al. (1995)
Linyphiidae	*Centromeritaconcinna* (Thorell, 1875)	Palaearctic (European)	Subboreal	litter	Esyunin et al. (1995)
Linyphiidae	*Centromerusarcanus* (O. Pickard-Cambridge, 1873)	Holarctic (Greenland-West Siberian)	Polyzonal	litter, moss	Esyunin et al. (1995)
Linyphiidae	*Centromerusclarus* (L. Koch, 1879)	Palaearctic (Trans-Siberian)	Boreal	litter, moss	Esyunin et al. (1995)
Linyphiidae	*Centromerusincilium* (L. Koch, 1881)	Palaearctic (European-West Siberian)	Temperate	litter, moss	Esyunin et al. (1995)
Linyphiidae	*Centromeruslevitarsis* (Simon, 1884)	Palaearctic (European-Middle Siberian)	Boreal	moss	Esyunin et al. (1995)
Linyphiidae	*Centromerussylvaticus* (Blackwall, 1841)	Holarctic (Circum-Holarctic)	Polyzonal	litter, moss	Esyunin et al. (1995)
Linyphiidae	*Ceratinellabrevipes* (Westring, 1851)	Palaearctic (Trans-Palaearctic)	Temperate	litter	Esyunin et al. (1995)
Linyphiidae	*Ceratinellabrevis* (Wider, 1834)	Palaearctic (Trans-Palaearctic)	Temperate	litter	Esyunin et al. (1995)
Linyphiidae	*Ceratinellamajor* Kulczyński, 1894	Palaearctic (European)	Temperate	litter	Ukhova et al. (2014a)
Linyphiidae	*Ceratinellascabrosa* (O. Pickard-Cambridge, 1871)	Palaearctic (European-West Siberian)	Temperate	litter	Esyunin et al. (1995)
Linyphiidae	*Ceratinellawideri* (Thorell, 1871)	Palaearctic (Trans-Palaearctic)	Subboreal	litter	Esyunin and Ukhova (2001)
Linyphiidae	*Cnephalocotesobscurus* (Blackwall, 1834)	Holarctic (Circum-Holarctic)	Temperate	herb	Esyunin et al. (1995)
Linyphiidae	*Decipiphantesdecipiens* (L. Koch, 1879)	Palaearctic (North European-Middle Siberian)	Boreal-mountain	litter, moss	Esyunin et al. (1995) (as *Lepthyphantes*)
Linyphiidae	*Dicymbiumnigrum* (Blackwall, 1834)	Palaearctic (West-Central-Palaearctic)	Temperate	litter	Esyunin and Efimik (1996a)
Linyphiidae	*Dicymbiumtibiale* (Blackwall, 1836)	Palaearctic (European-West Siberian)	Temperate	litter	Esyunin and Efimik (1996a)
Linyphiidae	*Diplocentriabidentata* (Emerton, 1882)	Holarctic (Circum-Holarctic)	Boreal	litter, moss	Esyunin et al. (1995)
Linyphiidae	*Diplocephaluspicinus* (Blackwall, 1841)	Palaearctic (West-Palaearctic)	Temperate	litter	Esyunin et al. (1995)
Linyphiidae	*Diplostylaconcolor* (Wider, 1834)	Holarctic (Circum-Holarctic)	Temperate	litter	Ukhova and Esyunin (1996)
Linyphiidae	*Dismodicusbifrons* (Blackwall, 1841)	Palaearctic (Trans-Palaearctic)	Polyzonal	herb, shrub	Ukhova et al. (2014a)
Linyphiidae	*Drapetiscasocialis* (Sundevall, 1833)	Palaearctic (Trans-Palaearctic)	Temperate	tree stems	Esyunin and Efimik (1996a)
Linyphiidae	*Entelecaraacuminata* (Wider, 1834)	Palaearctic (West-Central-Palaearctic)	Subboreal	herb, shrub, canopy	Esyunin and Ukhova (2001)
Linyphiidae	*Entelecaracongenera* (O. Pickard-Cambridge, 1879)	Palaearctic (European-Middle Siberian)	Boreal-mountain	canopy	**New data**
Linyphiidae	*Erigoneatra* Blackwall, 1833	Holarctic (Circum-Holarctic)	Polyzonal	ground, herb	Esyunin et al. (2021)
Linyphiidae	*Erigonellahiemalis* (Blackwall, 1841)	Palaearctic (European-West Siberian)	Temperate	litter, herb	Esyunin et al. (1995)
Linyphiidae	*Erigonellaignobilis* (O. Pickard-Cambridge, 1871)	Palaearctic (Trans-European Siberian)	Boreal-mountain	litter, herb	Esyunin et al. (1995)
Linyphiidae	*Estrandiagrandaeva* (Keyserling, 1886)	Holarctic (Circum-Holarctic)	Boreal	herb	Esyunin et al. (1995)
Linyphiidae	*Flagelliphantesbergstromi* (Schenkel, 1931)	Palaearctic (North European-Middle Siberian)	Boreal	litter	Esyunin et al. (1995) (as *Lepthyphantes*)
Linyphiidae	*Floroniabucculenta* (Clerck, 1757)	Palaearctic (Trans-Palaearctic)	Subboreal	herb, shrub	Esyunin (2005)
Linyphiidae	*Gnathonariumdentatum* (Wider, 1834)	Palaearctic (Trans-Palaearctic)	Temperate	herb	Esyunin et al. (1995)
Linyphiidae	*Gonatiumrubellum* (Blackwall, 1841)	Palaearctic (Trans-Palaearctic)	Temperate	herb	Esyunin et al. (1998)
Linyphiidae	*Gongylidiellumlatebricola* (O. Pickard-Cambridge, 1871)	Palaearctic (European-West Siberian)	Temperate	litter, moss	Esyunin et al. (1995)
Linyphiidae	*Helophorainsignis* (Blackwall, 1841)	Holarctic (Circum-Holarctic)	Temperate	herb	Esyunin and Efimik (1996a)
Linyphiidae	*Hilairaherniosa* (Thorell, 1875)	Holarctic (Circum-Holarctic)	Boreal-mountain	litter	Esyunin and Efimik (1996a)
Linyphiidae	*Hypselistesjacksoni* (O. Pickard-Cambridge, 1903)	Holarctic (Sub-Circum-Holarctic)	Temperate	herb	Esyunin et al. (1995)
Linyphiidae	*Improphantescomplicatus* (Emerton, 1882)	Holarctic (Circum-Holarctic)	Arcto-boreal	litter	**New data**
Linyphiidae	*Incestophanteskochiellus* (Strand, 1900)	Palaearctic (North European-Trans-Siberian)	Boreal	herb, canopy	Esyunin et al. (1995)
Linyphiidae	*Kaestneriadorsalis* (Wider, 1834)	Palaearctic (European-Middle Siberian)	Temperate	herb	Esyunin and Efimik (2000)
Linyphiidae	*Kaestneriapullata* (O. Pickard-Cambridge, 1863)	Holarctic (Circum-Holarctic)	Temperate	herb, shrub, canopy	Esyunin et al. (1995)
Linyphiidae	*Leptorhoptrumrobustum* (Westring, 1851)	Palaearctic (Trans-Palaearctic)	Arcto-boreal	litter, ground	Esyunin et al. (1995)
Linyphiidae	*Linyphiatriangularis* (Clerck, 1757)	Palaearctic (Trans-Palaearctic)	Temperate	herb, shrub	Ukhova et al. (2014a)
Linyphiidae	*Lophommapunctatum* (Blackwall, 1841)	Palaearctic (European-West Siberian)	Temperate	litter, moss	**New data**
Linyphiidae	*Macrargusmultesimus* (O. Pickard-Cambridge, 1875)	Holarctic (Circum-Holarctic)	Temperate	litter, moss	Esyunin and Ukhova (2001)
Linyphiidae	*Macrargusrufus* (Wider, 1834)	Palaearctic (European-West Siberian)	Temperate	litter	Esyunin et al. (1995)
Linyphiidae	*Marominutus* O. Pickard-Cambridge, 1907	Palaearctic (European)	Temperate	litter, moss	Esyunin et al. (1995)
Linyphiidae	*Maropansibiricus* Tanasevitch, 2006	Palaearctic (Trans-Siberian)	Boreal	litter	Esyunin et al. (1995) (as *Marosublestus*)
Linyphiidae	*Masosundevalli* (Westring, 1851)	Holarctic (Circum-Holarctic)	Polyzonal	litter	Ukhova et al. (2014a)
Linyphiidae	*Metopobactrusprominulus* (O. Pickard-Cambridge, 1873)	Holarctic (Sub-Circum Holarctic)	Temperate	litter, herb	Ukhova et al. (2014a)
Linyphiidae	*Micrargusherbigradus* (Blackwall, 1854)	Palaearctic (Trans-Palaearctic)	Temperate	litter	Esyunin et al. (1995)
Linyphiidae	*Micrargussubaequalis* (Westring, 1851)	Palaearctic (Trans-Palaearctic)	Temperate	litter	Esyunin et al. (1995)
Linyphiidae	*Microlinyphiapusilla* (Sundevall, 1830)	Holarctic (Circum-Holarctic)	Polyzonal	herb	Esyunin and Efimik (1996a)
Linyphiidae	*Micronetaviaria* (Blackwall, 1841)	Holarctic (Circum-Holarctic)	Temperate	litter	Esyunin and Efimik (1996a)
Linyphiidae	*Miniciamarginella* (Wider, 1834)	Palaearctic (Trans-Palaearctic)	Temperate	litter, herb	Esyunin and Efimik (1996a)
Linyphiidae	*Minyrioluspusillus* (Wider, 1834)	Palaearctic (Trans-European Siberian)	Boreal	litter, moss	Esyunin et al. (1995)
Linyphiidae	*Mughiphantescornutus* (Schenkel, 1927)	Palaearctic (European-Middle Siberian)	Boreal	litter	Ukhova et al. (2014a)
Linyphiidae	*Nerieneclathrata* (Sundevall, 1830)	Palaearctic (Trans-Palaearctic)	Temperate	herb, shrub	Esyunin et al. (2000)
Linyphiidae	*Nerieneemphana* (Walckenaer, 1841)	Holarctic (Sub-Circum-Holarctic)	Temperate	shrub, canopy	Esyunin and Efimik (1996a)
Linyphiidae	*Nerienemontana* (Clerck, 1757)	Palaearctic (Trans-Palaearctic)	Temperate	shrub, tree stems, canopy	Esyunin and Efimik (1996a)
Linyphiidae	*Nerieneradiata* (Walckenaer, 1841)	Holarctic (Circum-Holarctic)	Temperate	shrub, canopy	Ukhova and Esyunin (1996)
Linyphiidae	*Obscuriphantesobscurus* (Blackwall, 1841)	Palaearctic (European-WestSiberian)	Boreal-mountain	canopy	Esyunin et al. (1995) (as *Lepthyphantes*)
Linyphiidae	*Oedothoraxagrestis* (Blackwall, 1853)	Palaearctic (West-Central-Palaearctic)	Temperate	litter, ground	Ukhova and Esyunin (2006) (as *O.fuscus*)
Linyphiidae	*Oedothoraxapicatus* (Blackwall, 1850)	Palaearctic (West-Central-Palaearctic)	Polyzonal	litter, ground	Esyunin et al. (1995)
Linyphiidae	*Oedothoraxgibbosus* (Blackwall, 1841)	Palaearctic (European-West Siberian)	Temperate	litter, ground	Esyunin et al. (1995)
Linyphiidae	*Oedothoraxretusus* (Westring, 1851)	Palaearctic (Trans-Palaearctic)	Temperate	litter, ground	Ukhova et al. (2014a)
Linyphiidae	*Oreonetidesvaginatus* (Thorell, 1872)	Holarctic (Circum-Holarctic)	Boreal-mountain	litter, herb	Esyunin et al. (1995)
Linyphiidae	*Oryphantesangulatus* (O. Pickard-Cambridge, 1881)	Palaearctic (European-West Siberian)	Temperate	litter	Esyunin et al. (1995) (as *Lepthyphantesgeminus*)
Linyphiidae	*Palliduphantesalutacius* (Simon, 1884)	Palaearctic (European-West Siberian)	Boreal-mountain	litter	Esyunin and Efimik (1996a) (as *Lepthyphantespallidus*)
Linyphiidae	*Palliduphantesantroniensis* (Schenkel, 1933)	Palaearctic (European-West Siberian)	Boreal-mountain	litter	Esyunin and Ukhova (2011)
Linyphiidae	*Panamomopsdybowskii* (O. Pickard-Cambridge, 1873)	Palaearctic (North European-Middle Siberian)	Boreal	litter, moss	Esyunin et al. (1995)
Linyphiidae	*Pelecopsismengei* (Simon, 1884)	Holarctic (Circum-Holarctic)	Polyzonal	litter, moss	Esyunin et al. (2021)
Linyphiidae	*Pityohyphantesphrygianus* (C. L. Koch, 1836)	Palaearctic (Trans-Palaearctic)	Temperate	shrub, tree stems, canopy	Esyunin and Efimik (1996a)
Linyphiidae	*Pocadicnemispumila* (Blackwall, 1841)	Holarctic (Circum-Holarctic)	Temperate	litter	Esyunin et al. (1995)
Linyphiidae	*Poecilonetavariegata* (Blackwall, 1841)	Holarctic (Circum-Holarctic)	Boreal-mountain	herb, shrub, canopy	Esyunin and Efimik (2000)
Linyphiidae	*Porrhommamicrophthalmum* (O. Pickard-Cambridge, 1871)	Palaearctic (West-Central-Palaearctic)	Subboreal	litter	Esyunin and Efimik (1996a)
Linyphiidae	*Porrhommapallidum* Jackson, 1913	Palaearctic (Trans-Palaearctic)	Temperate	litter	Esyunin and Efimik (1996a)
Linyphiidae	*Porrhommapygmaeum* (Blackwall, 1834)	Palaearctic (Trans-Palaearctic)	Temperate	litter	Esyunin et al. (1996)
Linyphiidae	*Savigniabirostra* (Chamberlin & Ivie, 1947)	Holarctic (Trans-Siberian-West Nearctic)	Boreal	litter	Esyunin and Efimik (1996a) (as *S.nenilini*)
Linyphiidae	*Savigniaproducta* Holm, 1977	Palaearctic (North European-Middle Siberian)	Boreal	litter	Esyunin et al. (1998)
Linyphiidae	*Scotinotylusalpigena* (L. Koch, 1869)	Palaearctic (Trans-Palaearctic)	Boreal-mountain	litter	Esyunin et al. (1995)
Linyphiidae	*Semljicolalatus* (Holm, 1939)	Palaearctic (North European-Trans-Siberian)	Boreal	litter, moss	Esyunin et al. (1995) (as *Latithorax*)
Linyphiidae	*Semljicolathaleri* (Eskov, 1981)	Palaearctic (North European-Trans-Siberian)	Boreal-mountain	litter, moss	Esyunin et al. (1995) (as *Latithorax*)
Linyphiidae	*Stemonyphantesconspersus* (L. Koch, 1879)	Palaearctic (European-Middle Siberian)	Boreal-mountain	shrub	Esyunin and Ukhova (2011)
Linyphiidae	*Tallusiaexperta* (O. Pickard-Cambridge, 1871)	Palaearctic (Trans-European Siberian)	Temperate	litter, moss	Esyunin et al. (1995)
Linyphiidae	*Tapinocybabiscissa* (O. Pickard-Cambridge, 1873)	Palaearctic (European)	Subboreal	litter	Esyunin and Ukhova (2011)
Linyphiidae	*Tapinocybainsecta* (L. Koch, 1869)	Palaearctic (European-West Siberian)	Temperate	litter	Esyunin et al. (1995)
Linyphiidae	*Tapinopalongidens* (Wider, 1834)	Palaearctic (Amphi-Palaearctic)	Subboreal	litter	Esyunin et al. (1995)
Linyphiidae	*Taranucnussetosus* (O. Pickard-Cambridge, 1863)	Palaearctic (European-West Siberian)	Temperate	herb	Esyunin et al. (2021)
Linyphiidae	*Tenuiphantesalacris* (Blackwall, 1853)	Palaearctic (Trans-European Siberian)	Boreal-mountain	litter	Esyunin et al. (1995) (as *Lepthyphantes*)
Linyphiidae	*Tenuiphantescristatus* (Menge, 1866)	Palaearctic (European-West Siberian)	Temperate	litter	Esyunin et al. (1995) (as *Lepthyphantes*)
Linyphiidae	*Tenuiphantesmengei* (Kulczyński, 1887)	Palaearctic (Trans-Palaearctic)	Temperate	litter	Esyunin et al. (1996) (as *Lepthyphantes*)
Linyphiidae	*Tenuiphantesnigriventris* (L. Koch, 1879)	Palaearctic (North European-Trans-Siberian)	Boreal-mountain	litter	Esyunin et al. (1995) (as *Lepthyphantes*)
Linyphiidae	*Tenuiphantestenebricola* (Wider, 1834)	Palaearctic (European-West Siberian)	Temperate	litter	Esyunin et al. (1995) (as *Lepthyphantes*)
Linyphiidae	*Thyreosteniusparasiticus* (Westring, 1851)	Holarctic (Circum-Holarctic)	Temperate	litter	**New data**
Linyphiidae	*Tibioplusdiversus* (L. Koch, 1879)	Holarctic (Trans-European Siberian-West Nearctic)	Boreal-mountain	litter	Esyunin et al. (1995)
Linyphiidae	*Trematocephaluscristatus* (Wider, 1834)	Palaearctic (Trans-Palaearctic)	Temperate	canopy	Esyunin and Ukhova (2011)
Linyphiidae	*Walckenaeriaantica* (Wider, 1834)	Palaearctic (Trans-Palaearctic)	Temperate	litter	Esyunin et al. (1998)
Linyphiidae	*Walckenaeriaatrotibialis* (O. Pickard-Cambridge, 1878)	Holarctic (Circum-Holarctic)	Temperate	litter	Esyunin and Efimik (2000)
Linyphiidae	*Walckenaeriacuspidata* Blackwall, 1833	Palaearctic (Trans-European Siberian)	Arcto-boreal-mountain	litter	Esyunin et al. (1995)
Linyphiidae	*Walckenaeriakarpinskii* (O. Pickard-Cambridge, 1873)	Holarctic (Circum-Holarctic)	Arcto-boreal-mountain	litter, moss	Esyunin and Efimik (1996a)
Linyphiidae	*Walckenaeriakochi* (O. Pickard-Cambridge, 1873)	Holarctic (Sub-Circum-Holarctic)	Temperate	litter	**New data**
Linyphiidae	*Walckenaerialepida* (Kulczyński, 1885)	Holarctic (Circum-Holarctic)	Temperate	litter	Esyunin and Efimik (1996a)
Linyphiidae	*Walckenaeriamitrata* (Menge, 1868)	Palaearctic (European)	Temperate	litter, moss	Ukhova and Esyunin (2006)
Linyphiidae	*Walckenaerianodosa* O. Pickard-Cambridge, 1873	Holarctic (Sub-Circum-Holarctic)	Temperate	litter	Esyunin et al. (1995)
Linyphiidae	*Walckenaerianudipalpis* (Westring, 1851)	Palaearctic (Trans-European Siberian)	Temperate	litter, moss	Esyunin et al. (1995)
Linyphiidae	*Walckenaeriaobtusa* Blackwall, 1836	Palaearctic (Amphi-Palaearctic)	Temperate	litter	Esyunin et al. (1995)
Linyphiidae	*Walckenaeriapicetorum* (Palmgren, 1976)	Palaearctic (North European-Trans-Siberian)	Boreal	litter, moss	Esyunin et al. (1995)
Linyphiidae	*Walckenaeriaunicornis* O. Pickard-Cambridge, 1861	Palaearctic (European-West Siberian)	Temperate	litter, moss	Esyunin et al. (1995)
Linyphiidae	*Walckenaeriavigilax* (Blackwall, 1853)	Holarctic (Sub-Circum-Holarctic)	Temperate	litter	Ukhova et al. (2014a)
Linyphiidae	*Wubanoidesuralensis* (Pakhorukov, 1981)	Palaearctic (East European-West Siberian)	Boreal-mountain	rocks, tree stems	Ukhova and Esyunin (1996)
Linyphiidae	*Zornellacultrigera* (L. Koch, 1879)	Palaearctic (North European-Trans-Siberian)	Boreal	litter, moss	Esyunin et al. (1995)
Liocranidae	*Agroecabrunnea* (Blackwall, 1833)	Palaearctic (Trans-Palaearctic)	Temperate	ground	Esyunin et al. (1998)
Liocranidae	*Agroecalusatica* (L. Koch, 1875)	Palaearctic (West-Palaearctic)	Subboreal	ground	**New data**
Liocranidae	*Agroecaproxima* (O. Pickard-Cambridge, 1871)	Palaearctic (European-West Siberian)	Subboreal	ground	Esyunin et al. (1998)
Lycosidae	*Acantholycosalignaria* (Clerck, 1757)	Palaearctic (Trans-European Siberian)	Temperate	tree stems	Ukhova and Esyunin (1996)
Lycosidae	*Acantholycosanorvegica* (Thorell, 1872)	Palaearctic (North European-Trans-Siberian)	Boreal-mountain	ground, rocks	Esyunin and Efimik (1996a)
Lycosidae	*Alopecosapinetorum* (Thorell, 1856)	Palaearctic (West-Palaearctic)	Boreal-mountain	moss, ground	Esyunin and Efimik (1995)
Lycosidae	*Alopecosapulverulenta* (Clerck, 1757)	Palaearctic (Trans-Palaearctic)	Temperate	ground	Esyunin and Efimik (1996a)
Lycosidae	*Alopecosataeniata* (C. L. Koch, 1835)	Palaearctic (European-West Siberian)	Boreal	ground	Esyunin and Efimik (1996a) (as *A.aculeata*)
Lycosidae	*Hygrolycosarubrofasciata* (Ohlert, 1865)	Palaearctic (West-Central-Palaearctic)	Subboreal	litter, moss	Esyunin and Ukhova (2001)
Lycosidae	*Pardosaagrestis* (Westring, 1861)	Palaearctic (West-Palaearctic)	Polyzonal	ground	Esyunin and Efimik (2000)
Lycosidae	*Pardosaamentata* (Clerck, 1757)	Palaearctic (West-Palaearctic)	Polyzonal	ground	Esyunin and Efimik (1996a)
Lycosidae	*Pardosafulvipes* (Collett, 1876)	Palaearctic (European-West Siberian)	Temperate	ground	Esyunin and Efimik (1995)
Lycosidae	*Pardosalugubris* (Walckenaer, 1802)	Palaearctic (West-Palaearctic)	Temperate	ground	Esyunin and Efimik (1996a)
Lycosidae	*Pardosapaludicola* (Clerck, 1757)	Palaearctic (West-Palaearctic)	Subboreal	ground	Esyunin and Ukhova (2011)
Lycosidae	*Pardosaprativaga* (L. Koch, 1870)	Palaearctic (West-Central-Palaearctic)	Temperate	ground	**New data**
Lycosidae	*Pardosariparia* (C. L. Koch, 1833)	Palaearctic (Trans-Palaearctic)	Temperate	ground, herb	Esyunin and Efimik (1996a)
Lycosidae	*Pardosasphagnicola* (Dahl, 1908)	Palaearctic (European-WestSiberian)	Boreal	ground	Esyunin and Ukhova (2011)
Lycosidae	*Piratapiraticus* (Clerck, 1757)	Holarctic (Circum-Holarctic)	Polyzonal	moss and water surface	Esyunin and Efimik (1996a)
Lycosidae	*Piratulahygrophila* (Thorell, 1872)	Palaearctic (West-Central-Palaearctic)	Temperate	ground	Esyunin and Efimik (1996a) (as *Pirata*)
Lycosidae	*Piratulauliginosa* (Thorell, 1856)	Palaearctic (European-West Siberian)	Temperate	moss	Esyunin and Efimik (1995) (as *Pirata*)
Lycosidae	*Trochosaruricola* (De Geer, 1778)	Palaearctic (Trans-Palaearctic)	Polyzonal	ground	Esyunin and Ukhova (2001)
Lycosidae	*Trochosaspinipalpis* (F. O. Pickard-Cambridge, 1895)	Palaearctic (Amphi-Palaearctic)	Temperate	moss, ground	Esyunin and Efimik (2000)
Lycosidae	*Trochosaterricola* Thorell, 1856	Holarctic (Circum-Holarctic)	Temperate	ground	Esyunin and Efimik (1996a)
Lycosidae	*Xerolycosanemoralis* (Westring, 1861)	Palaearctic (Trans-Palaearctic)	Temperate	ground	Esyunin and Ukhova (2001)
Mimetidae	*Erofurcata* (Villers, 1789)	Palaearctic (Trans-Palaearctic)	Temperate	herb, shrub, canopy	Esyunin and Efimik (1996a)
Miturgidae	*Zoraspinimana* (Sundevall, 1833)	Palaearctic (Trans-Palaearctic)	Temperate	ground	Esyunin and Ukhova (2011)
Philodromidae	*Philodromuscespitum* (Walckenaer, 1802)	Holarctic (Circum-Holarctic)	Polyzonal	herb, shrub, canopy	Esyunin and Efimik (1995)
Philodromidae	*Philodromusemarginatus* (Schrank, 1803)	Palaearctic (Trans-Palaearctic)	Temperate	shrub, tree stems	Esyunin and Ukhova (2001)
Philodromidae	*Philodromusfuscomarginatus* (De Geer, 1778)	Palaearctic (Trans-European Siberian)	Temperate	tree stems	Ukhova and Esyunin (2006)
Philodromidae	*Philodromusmargaritatus* (Clerck, 1757)	Palaearctic (Trans-Palaearctic)	Temperate	tree stems	Esyunin and Efimik (1996a)
Philodromidae	*Thanatusformicinus* (Clerck, 1757)	Holarctic (Circum-Holarctic)	Polyzonal	ground, herb	Ukhova and Esyunin (1996)
Philodromidae	*Thanatusstriatus* C. L. Koch, 1845	Holarctic (Circum-Holarctic)	Polyzonal	ground	Esyunin (2015)
Philodromidae	*Tibellusoblongus* (Walckenaer, 1802)	Holarctic (Circum-Holarctic)	Polyzonal	herb	Esyunin and Ukhova (2001)
Phrurolithidae	*Phrurolithusfestivus* (C. L. Koch, 1835)	Palaearctic (Trans-Palaearctic)	Subboreal	litter, ground	Esyunin and Ukhova (2001)
Pisauridae	*Dolomedesfimbriatus* (Clerck, 1757)	Palaearctic (Trans-Palaearctic)	Temperate	herb	Esyunin and Efimik (1996a)
Pisauridae	*Pisauramirabilis* (Clerck, 1757)	Palaearctic (West-Central-Palaearctic)	Subboreal-subarid	herb	Esyunin et al. (1998)
Salticidae	*Dendryphantesrudis* (Sundevall, 1833)	Palaearctic (Trans-European Siberian)	Temperate	herb, shrub	Esyunin and Efimik (1996a)
Salticidae	*Euophrysfrontalis* (Walckenaer, 1802)	Palaearctic (Trans-Palaearctic)	Temperate	litter, ground	**New data**
Salticidae	*Evarchaarcuata* (Clerck, 1757)	Palaearctic (Trans-Palaearctic)	Temperate	herb	Esyunin and Ukhova (2001)
Salticidae	*Evarchafalcata* (Clerck, 1757)	Palaearctic (West-Central-Palaearctic)	Temperate	herb	Esyunin and Efimik (1996a)
Salticidae	*Evarchalaetabunda* (C. L. Koch, 1846)	Palaearctic (Trans-Palaearctic)	Temperate	herb	Esyunin and Ukhova (2001)
Salticidae	*Heliophanuscamtschadalicus* Kulczyński, 1885	Palaearctic (North European-Trans-Siberian)	Boreal	herb	Esyunin and Efimik (1995) (as *H.dampfi*)
Salticidae	*Heliophanusdubius* C. L. Koch, 1835	Palaearctic (Trans-Palaearctic)	Temperate	herb	Esyunin and Ukhova (2001)
Salticidae	*Marpissapomatia* (Walckenaer, 1802)	Palaearctic (Trans-Palaearctic)	Temperate	herb	Esyunin and Ukhova (2001)
Salticidae	*Neonreticulatus* (Blackwall, 1853)	Holarctic (Sub-Circum-Holarctic)	Temperate	litter	Esyunin and Efimik (1996a)
Salticidae	*Pseudeuophryserratica* (Walckenaer, 1826)	Palaearctic (Trans-Palaearctic)	Temperate	litter	Esyunin and Efimik (1995) (as *Euophrys*)
Salticidae	*Salticuscingulatus* (Panzer, 1797)	Palaearctic (Trans-Palaearctic)	Temperate	tree stems	Esyunin (2015)
Salticidae	*Sibianorlarae* Logunov, 2001	Palaearctic (Trans-European Siberian)	Boreal	ground, herb	Ukhova et al. (2014a)
Salticidae	*Synagelesvenator* (Lucas, 1836)	Palaearctic (Trans-Palaearctic)	Temperate	herb, shrub	Esyunin and Efimik (1995)
Salticidae	*Talaverathorelli* (Kulczyński, 1891)	Palaearctic (European-Middle Siberian)	Boreal-mountain	ground	**New data**
Sparassidae	*Micrommatavirescens* (Clerck, 1757)	Palaearctic (Trans-Palaearctic)	Temperate	herb	Esyunin and Efimik (1996a)
Tetragnathidae	*Metellinamengei* (Blackwall, 1869)	Palaearctic (West-Palaearctic)	Temperate	herb	Esyunin and Efimik (1996a)
Tetragnathidae	*Metellinamerianae* (Scopoli, 1763)	Palaearctic (West-Palaearctic)	Temperate	rocks, caves, tree stems	Esyunin and Efimik (1996a)
Tetragnathidae	*Metellinasegmentata* (Clerck, 1757)	Palaearctic (Trans-Palaearctic)	Temperate	herb	Esyunin and Efimik (1996a)
Tetragnathidae	*Pachygnathadegeeri* Sundevall, 1830	Palaearctic (Trans-Palaearctic)	Polyzonal	ground, herb	Esyunin and Efimik (1996a)
Tetragnathidae	*Pachygnathalisteri* Sundevall, 1830	Palaearctic (Trans-European Siberian)	Temperate	ground, herb	Esyunin and Efimik (1996a)
Tetragnathidae	*Tetragnathadearmata* Thorell, 1873	Holarctic (Circum-Holarctic)	Temperate	shrub	**New data**
Tetragnathidae	*Tetragnathaextensa* (Linnaeus, 1758)	Holarctic (Circum-Holarctic)	Polyzonal	herb, shrub, canopy	Ukhova and Esyunin (1996)
Tetragnathidae	*Tetragnathapinicola* L. Koch, 1870	Palaearctic (Trans-Palaearctic)	Polyzonal	herb, shrub	Esyunin and Efimik (1995)
Theridiidae	*Canalidionmontanum* (Emerton, 1882)	Holarctic (Circum-Holarctic)	Boreal	tree stems, canopy	Esyunin and Efimik (1995) (as *Theridion*)
Theridiidae	*Enoplognathaovata* (Clerck, 1757)	Holarctic (Circum-Holarctic)	Polyzonal	herb, shrub	Ukhova and Esyunin (1996)
Theridiidae	*Euryopisflavomaculata* (C. L. Koch, 1836)	Palaearctic (Trans-Palaearctic)	Temperate	litter	Esyunin (2005)
Theridiidae	*Lasaeolaprona* (Menge, 1868)	Holarctic (Nearctic-West Siberian)	Subboreal	herb	Ukhova et al. (2014a)
Theridiidae	*Neottiurabimaculata* (Linnaeus, 1767)	Holarctic (Circum-Holarctic)	Temperate	herb	Esyunin and Ukhova (2001)
Theridiidae	*Ohlertidionohlerti* (Thorell, 1870)	Holarctic (Circum-Holarctic)	Boreal-mountain	tree stems, canopy	Esyunin and Efimik (1995) (as *Achaearanea*)
Theridiidae	*Phyllonetaimpressa* (L. Koch, 1881)	Holarctic (Circum-Holarctic)	Polyzonal	herb, shrub	Esyunin and Efimik (1996a) (as *Theridion*)
Theridiidae	*Robertusarundineti* (O. Pickard-Cambridge, 1871)	Palaearctic (West-Central-Palaearctic)	Temperate	litter	Esyunin and Efimik (1995)
Theridiidae	*Robertuslividus* (Blackwall, 1836)	Holarctic (Trans-Palaearctic-West Nearctic)	Temperate	litter	Esyunin and Efimik (1995)
Theridiidae	*Rugathodesaurantius* (Emerton, 1915)	Holarctic (Circum-Holarctic)	Boreal	herb, shrub	Esyunin and Efimik (1995) (as *Rugathodesinstabile*)
Theridiidae	*Steatodabipunctata* (Linnaeus, 1758)	Palaearctic (Trans-Palaearctic)	Temperate	tree stems and outside buildings	Esyunin and Efimik (1996a)
Theridiidae	*Theridionpictum* (Walckenaer, 1802)	Holarctic (Circum-Holarctic)	Subboreal-subarid	herb, shrub	Esyunin and Efimik (1996a)
Theridiidae	*Theridionvarians* Hahn, 1833	Holarctic (Sub-Circum-Holarctic)	Temperate	tree stems	Esyunin and Efimik (1996a)
Theridiidae	*Thymoitesbellissimus* (L. Koch, 1879)	Palaearctic (North European-Trans-Siberian)	Boreal	rocks	Esyunin and Efimik (1996a)
Theridiidae	*Yunohamellapalmgreni* (Marusik & Tsellarius, 1986)	Palaearctic (East European-West Siberian)	Boreal	canopy	Esyunin and Efimik (1995) (as *Theridion*)
Theridiidae	*Yunohamellaserpatusa* (Guan & Zhu, 1993)	Palaearctic (Trans-Siberian)	Subboreal	canopy	Esyunin and Efimik (1996a)
Thomisidae	*Misumenavatia* (Clerck, 1757)	Holarctic (Circum-Holarctic)	Polyzonal	herb	Ukhova and Esyunin (1996)
Thomisidae	*Ozyptilaatomaria* (Panzer, 1801)	Palaearctic (Trans-Palaearctic)	Temperate	moss, ground	Esyunin and Ukhova (2011)
Thomisidae	*Ozyptilapraticola* (C. L. Koch, 1837)	Palaearctic (West-Central-Palaearctic)	Temperate	litter	Esyunin and Efimik (1996a)
Thomisidae	*Ozyptilatrux* (Blackwall, 1846)	Palaearctic (Trans-European Siberian)	Temperate	litter	Esyunin and Efimik (1996a)
Thomisidae	*Xysticusaudax* (Schrank, 1803)	Palaearctic (Trans-Palaearctic)	Polyzonal	herb	Esyunin and Efimik (1996a)
Thomisidae	*Xysticusbifasciatus* C. L. Koch, 1837	Palaearctic (West-Central-Palaearctic)	Temperate	ground, herb	Esyunin and Efimik (1996a)
Thomisidae	*Xysticuscristatus* (Clerck, 1757)	Palaearctic (Trans-Palaearctic)	Polyzonal	herb	Esyunin and Efimik (1996a)
Thomisidae	*Xysticuskochi* Thorell, 1872	Palaearctic (West-Palaearctic)	Subboreal-subarid	herb	**New data**
Thomisidae	*Xysticuslineatus* (Westring, 1851)	Palaearctic (European-West Siberian)	Temperate	herb	Esyunin and Ukhova (2001)
Thomisidae	*Xysticusluctuosus* (Blackwall, 1836)	Holarctic (Circum-Holarctic)	Temperate	ground, herb	Esyunin and Ukhova (2001)
Thomisidae	*Xysticusobscurus* Collett, 1877	Holarctic (Circum-Holarctic)	Boreal-mountain	ground, herb	Esyunin and Ukhova (2011)
Thomisidae	*Xysticusslovacus* Svatoň, Pekár & Prídavka, 2000	Palaearctic (EastEuropean)	Temperate	herb	Esyunin and Efimik (1995) (as *X.ukrainicus*)
Thomisidae	*Xysticusulmi* (Hahn, 1831)	Palaearctic (West-Central-Palaearctic)	Temperate	herb	Esyunin and Efimik (1996a)
Thomisidae	*Xysticusviduus* Kulczyński, 1898	Palaearctic (European-West Siberian)	Polyzonal	ground, herb	Esyunin et al. (1998)
